# Neurosteroids in Adult Hippocampus of Male and Female Rodents: Biosynthesis and Actions of Sex Steroids

**DOI:** 10.3389/fendo.2018.00183

**Published:** 2018-04-23

**Authors:** Yasushi Hojo, Suguru Kawato

**Affiliations:** ^1^Department of Biochemistry, Faculty of Medicine, Saitama Medical University, Moroyama, Saitama, Japan; ^2^Department of Biophysics and Life Sciences, Graduate School of Arts and Sciences, The University of Tokyo, Tokyo, Japan; ^3^Department of Urology, Graduate School of Medicine, Juntendo University, Tokyo, Japan; ^4^Department of Cognitive Neuroscience, Faculty of Pharma-Science, Teikyo University, Tokyo, Japan

**Keywords:** hippocampus, neurosteroids, estradiol, testosterone, dihydrotestosterone, estrous cycle, synaptic plasticity, neurogenesis

## Abstract

The brain is not only the target of steroid hormones but also is able to locally synthesize steroids *de novo*. Evidence of the local production of steroids in the brain has been accumulating in various vertebrates, including teleost fish, amphibia, birds, rodents, non-human primates, and humans. In this review, we mainly focus on the local production of sex steroids in the hippocampal neurons of adult rodents (rats and mice), a center for learning and memory. From the data of the hippocampus of adult male rats, hippocampal principal neurons [pyramidal cells in CA1–CA3 and granule cells in dentate gyrus (DG)] have a complete system for biosynthesis of sex steroids. Liquid chromatography with tandem-mass-spectrometry (LC-MS/MS) enabled us to accurately determine the levels of hippocampal sex steroids including 17β-estradiol (17β-E2), testosterone (T), and dihydrotestosterone (DHT), which are much higher than those in blood. Next, we review the steroid synthesis in the hippocampus of female rats, since previous knowledge had been biased toward the data from males. Recently, we clarified that the levels of hippocampal steroids fluctuate in adult female rats across the estrous cycle. Accurate determination of hippocampal steroids at each stage of the estrous cycle is of importance for providing the account for the fluctuation of female hippocampal functions, including spine density, long-term potentiation (LTP) and long-term depression (LTD), and learning and memory. These functional fluctuations in female had been attributed to the level of circulation-derived steroids. LC-MS/MS analysis revealed that the dendritic spine density in CA1 of adult female hippocampus correlates with the levels of hippocampal progesterone and 17β-E2. Finally, we introduce the direct evidence of the role of hippocampus-synthesized steroids in hippocampal function including neurogenesis, LTP, and memory consolidation. Mild exercise (2 week of treadmill running) elevated synthesis of DHT in the hippocampus, but not in the testis, of male rats, resulting in enhancement of neurogenesis in DG. Concerning synaptic plasticity, hippocampus-synthesized E2 is required for LTP induction, whereas hippocampus-synthesized DHT is required for LTD induction. Furthermore, hippocampus-synthesized E2 is involved in memory consolidation tested by object recognition and object placement tasks, both of which are hippocampus-dependent.

## Introduction

Extensive evidence has been accumulated that the systems of local steroid synthesis exist in the organs other than gonads and adrenal since 1980s (1, 2). Local production of steroids in the brain has been investigated in various vertebrates, including teleost fish (3, 4), amphibia (5–7), birds (8, 9), rodents (10–14), non-human primates, and humans (15–17).

For clinical purposes, the importance of neurosteroids is increasing. Because of the limitation to invade human brain tissues, quantitative determination of steroids in cerebrospinal fluid (CSF) has been applied to detect the alteration of the allopregnanolone (Allo) level under physiological/pathological conditions, including epilepsy (18, 19), and reproductive mood disorders (20, 21).

Sex steroids including 17β-estradiol (17β-E2), testosterone (T), and dihydrotestosterone (DHT) are also synthesized in the brain. In this review, we mainly focus on the local production of sex steroids, particularly, E2, T, and DHT in the hippocampal neurons of adult rodents (rats and mice).

In addition to the genomic effects, sex steroids modulate neural functions in a rapid/non-genomic manner [reviewed in Ref. (22)]. Using the hippocampal slices of rodents, rapid effects of sex steroids have been extensively investigated. E2 modulates long-term potentiation (LTP) (23, 24) and long-term depression (LTD) (25, 26) in CA1 synapses. E2 induces LTP in CA1 under the weak theta burst stimulation (weak-TBS), which is not strong enough to induce LTP alone (27). Exogenous application of E2, T, and DHT to rat hippocampal slices, rapidly increases dendritic spines in CA1 pyramidal cells (27, 28).

Using exogenous application of steroids, these investigations demonstrated that rapid effects of sex steroids are mediated through estrogen receptors (ERα and ERβ) or androgen receptors (AR), located at the pre/post synapses (25, 28–30), followed by the activation of kinases which phosphorylate the molecules essential for synaptic plasticity. Upon LTP-induction, E2 drives src tyrosine kinase and the extracellular signal-related protein kinase/MAPK (Erk MAPK), resulting in phosphorylation of NMDA receptor (23). In case of E2-induced LTP by weak-TBS, Erk MAPK, PKA, PKC, PI3K, and CaMkII phosphorylate NR2B subunit (27). In addition to postsynaptic modulation, E2 also activates ERs in presynapses, resulting in potentiation of glutamate release (31) or disinhibition of GABAergic axon terminal (32). Concerning spinogenesis, E2, T, or DHT drives Erk MAPK, p38 MAPK, PKA, PKC, PI3K, and LIMK (27, 28), which may phosphorylate cortactin (33, 34) and cofilin (35, 36), leading to actin polymerization and spinogenesis.

**Figure 1:**
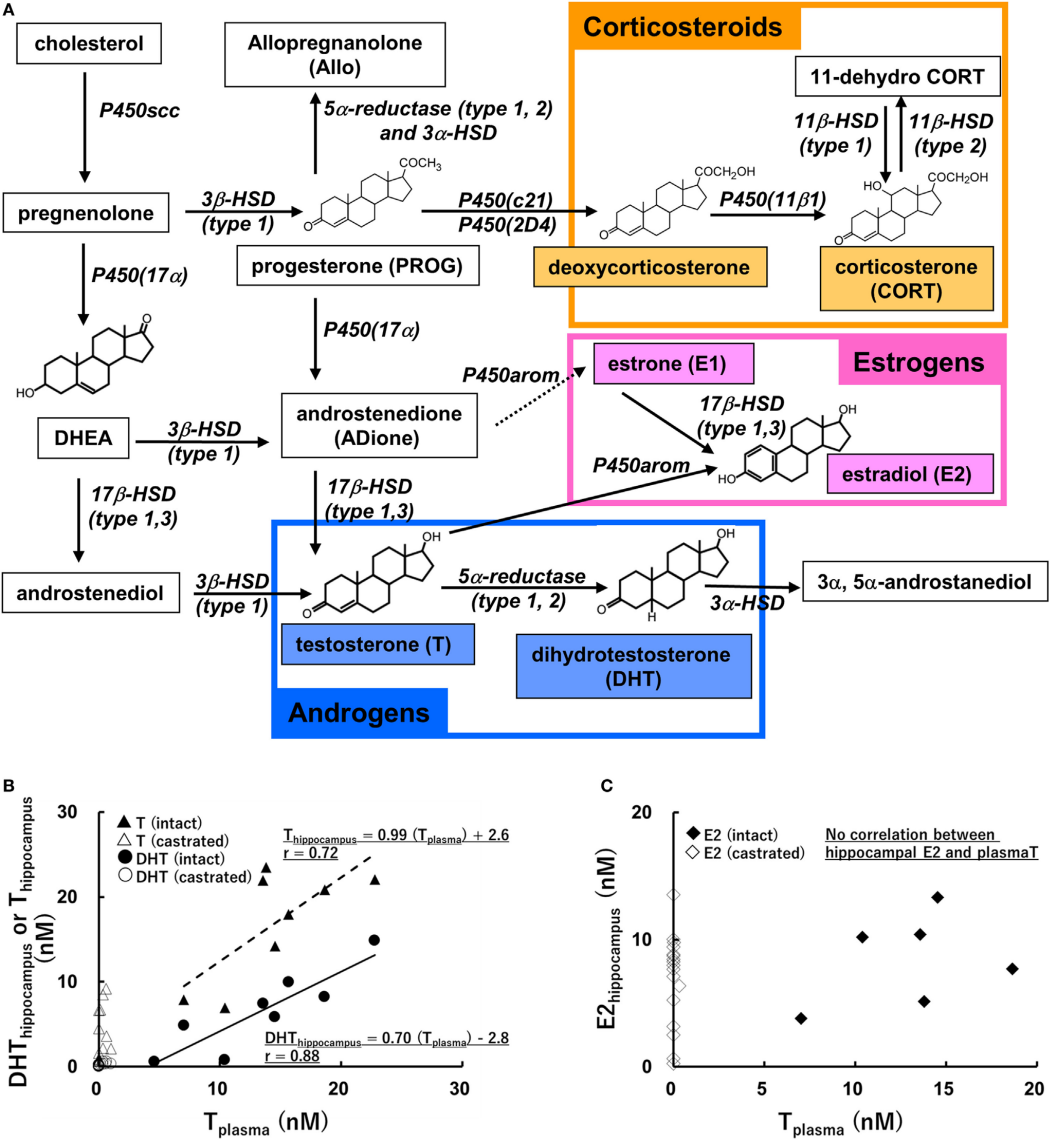
**(A)** Biosynthetic pathway of steroids in rat hippocampus [modified from Ref. (11)]. Estrogens, androgens, and corticosteroids are represented with pink, blue, and orange boxes, respectively. **(B,C)** Relationship between the level of plasma T and those of hippocampal androgens [T and dihydrotestosteron (DHT)] **(A)**, or that of hippocampal E2 **(B)**. Vertical axis represents hippocampal steroids and horizontal axis represents plasma T, a precursor of DHT and E2. The regression lines and Pearson’s “*r*” are indicated [modified from Ref. (37)].

Do the effects of hippocampus-synthesized E2, T and DHT share the common mechanism described above? It is difficult, however, to directly demonstrate the roles of hippocampus-synthesized E2, T, and DHT, because of the supply of E2, T, and DHT from testis or ovary. It is necessary to perform the experiment under the depletion of circulation-derived E2, T, and DHT, although the possibility is not excluded that peripherally produced precursors (e.g., pregnenolone and progesterone) convert into E2, T, or DHT in the hippocampus (Figure 1A). Several investigations are introduced in the Section “Physiological Roles of Hippocampus-Synthesized Steroids.”

## Steroid Biosynthesis in the Hippocampus

### Steroid Biosynthesis in the Hippocampus of Adult Male Rodents

Until about 15 years ago, it had not been elucidated whether adult hippocampal neurons have a complete system for synthesis of sex hormones (from cholesterol to androgens or estrogens) since P450(17α), which is required for synthesis of dehydroepiandrosterone (DHEA) from pregnenolone (PREG), had been thought to be absent in the brain of mammals. Any effort to demonstrate the existence of P450(17α) or its enzymatic activity had been unsuccessful (38–41) despite the presence of DHEA in the rodent brain even after castration (1, 2).

By using the hippocampus of adult male rats, the localization of P450(17α) in the principal neurons [pyramidal cells in CA1–CA3 and granule cells in dentate gyrus (DG)] was demonstrated (42, 43). Immunohistochemistry and *in situ* hybridization studies revealed that StAR and other enzymes, including P450scc, 3β-hydroxysteroid dehydrogenase (3β-HSD), 17β-HSD, 5α-reductase (types 1 and 2), and P450arom, are also localized in the hippocampal principal neurons of adult male rats and mice (37, 42–48). Studies with radioactive steroids directly demonstrated synthesis of PREG, DHEA, T, DHT, and E2 in slices or cultured hippocampal neurons from adult male rats in early 2000s (42, 43, 47, 49). These results suggest that complete systems for steroidogenesis exist in the hippocampal neurons of adult male rodents.

Interestingly, an electron microscopic (EM) analysis revealed synaptic localization of steroidogenic enzymes including P450(17α), P450arom, and 3β-HSD in the hippocampus of adult male rats (42, 50), implying the synaptic synthesis of sex steroids. EM and Western immunoblot analysis revealed localization of steroid receptors including ERα, ERβ, AR, and progesterone receptor (PR) in the hippocampal synapses of rodents (25, 28–30, 51, 52).

Although these results give information about the potential to synthesize steroids in the hippocampus, it remains unclear whether hippocampus-synthesized steroids are effective enough to modulate hippocampal functions. To answer this question, it is necessary to quantitatively determine the concentration of steroids in the hippocampus. From early 2000s, quantitative determination of steroids, such as PREG, DHEA, and T in brain with mass-spectrometry (MS) began to emerge (53–58). The presence of pregnenolone sulfate (PREGS) in the brain of mammals had been a matter of debate (59).

To detect small amounts of steroids in the brain, purification of samples and selection of appropriate derivatization reagents are indispensable. Extracts from brain tissue contain various kinds of impurities (lipids and other steroids) which mask derivatization and ionization of the steroid of interest, resulting in decrease of detection efficiency of MS. Purification of the extracts by hybrid-SPE cartridges before LC–ESI-MS/MS enabled the detection of PREGS in the rat hippocampus (60). Concerning the detection of sex steroids, we removed impurities from hippocampal extracts and separated into fractions containing an individual steroid, with C18 column and normal phase HPLC before derivatization. Next, picolinoyl-derivatization was selected for steroids of interest (E2, T, DHT, and E1) to increase ionization efficiency (61, 62). Concerning E2, further derivatization with pentafluorobenzyl was performed to elevate volatility. In combination with these improvements, LC-MS/MS enabled us to quantitatively determine the concentration of E2, T, DHT, and E1 in the hippocampus, with high accuracy and reproducibility (37). Caruso and collaborators also determined the levels of steroids including E2, T, and DHT in rat hippocampus, with LC-MS/MS methods (63). These results support that the significant amount of sex steroids exists in the hippocampus.

Correlation between the level of hippocampal androgen (T and DHT) and that of plasma T (Figure 1) was observed (37, 64). Hippocampal estrogen (E2), however, did not correlate with plasma T (Figure 1) (37, 64). Using male and female rats, Melcangi’s laboratory extensively analyzed the correlation of steroid levels among plasma, CSF and various brain regions (63). According to this work, the levels of E2, T, and DHT in the hippocampus or CSF positively correlated with those in plasma, but no significant correlation of E2 level was observed between in the hippocampus and CSF (63).

### Synthesis and Fluctuation of Steroids in the Hippocampus of Adult Female Rodents

Equally important is to clarify whether local steroid production occurs in female hippocampus, because sex hormones have a great impact on functions of female hippocampus [reviewed in Ref. (65)]. The knowledge of the hippocampus-synthesized steroids, however, had been biased toward the data from males (11–13) because of the estrous cycle in female animals. In case of rats and mice, estrous cycle comprises of four stages [proestrus: Pro, estrus: Est, diestrus1 (also called metestrus): D1, and diestrus2 (also called diestrus): D2], and each stage switches in 1 day in this order (66). Hippocampal functions such as spatial memory (67–71), LTP (72, 73), and spine/synapse density (74–78), fluctuate across the estrous cycle. To investigate hippocampal steroid synthesis in female rodents, therefore, fourfold as much data as those of male must be acquired.

LC-MS/MS analysis revealed the accurate concentrations of progesterone (PROG), androstenedione (ADione), T, E1, and E2 in the hippocampus of adult female rats at each stage of estrous cycle (Table 1) (79). The levels of plasma steroids exhibit typical estrous cycle dependent changes, in agreement with the previous study (80). The level of hippocampal E2 highly correlates with that of plasma E2, in agreement with other study (63). Concerning the correlation of PROG level between in hippocampus and in plasma, our data exhibit highly positive correlation, whereas others have no correlation (63). It may be due to the difference of samples used for calculation of correlations, rats of both sexes (male and diestrus female) in Caruso et al. (63) and only female (all four stages of the estrous cycle) in our study (79).

**Table 1:** Mass spectrometric analysis of the concentration of steroids in the hippocampus and plasma of adult rats.

	Male	Female^b^				
		Proestrus	Estrus	Diestrus1	Diestrus2	OVX
**(A) Hippocampus^a^**						
17β-E2	8.4^c^ (*n*^d^ = 6)	4.3 (*n* = 6)	1.0 (*n* = 4)	0.51 (*n* = 3)	0.67 (*n* = 4)	0.70 (*n* = 4)
T	16.9 (*n* = 8)	1.1 (*n* = 12)	2.3 (*n* = 4)	1.3 (*n* = 3)	1.2 (*n* = 4)	0.17 (*n* = 4)
Dihydrotestosteron (DHT)	6.6 (*n* = 8)	0.62 (*n* = 7)				
Progesterone (PROG)	14.6 (*n* = 4)	55.7 (*n* = 4)	40.7 (*n* = 4)	87.0 (*n* = 3)	48.0 (*n* = 4)	24.5 (*n* = 5)
Androstenedione	1.5 (*n* = 4)	1.6 (*n* = 4)	0.7 (*n* = 4)	1.1 (*n* = 4)	0.83 (*n* = 4)	
E1	0.015 (*n* = 4)	0.36 (*n* = 4)	0.045 (*n* = 4)	0.05 (*n* = 4)	0.10 (*n* = 4)	0.025 (*n* = 3)
Allopregnanolone	1.0 (*n* = 3)	16.4 (*n* = 3)				

**(B) Plasma**						
17β-E2	0.014 (*n* = 5)	0.111 (*n* = 6)	0.017 (*n* = 6)	0.009 (*n* = 5)	0.029 (*n* = 6)	0.005 (*n* = 5)
T	14.6 (*n* = 8)	0.10 (*n* = 4)	0.013 (*n* = 4)	0.020 (*n* = 3)	0.06 (*n* = 4)	0.005 (*n* = 5)
DHT	0.63 (*n* = 8)					
PROG	6.8 (*n* = 4)	20.5 (*n* = 4)	16.7 (*n* = 4)	51.6 (*n* = 3)	24.1 (*n* = 4)	10.1 (*n* = 5)
Androstenedione	0.61 (*n* = 4)	1.0 (*n* = 4)	0.06 (*n* = 4)	0.119 (*n* = 4)	0.33 (*n* = 4)	
E1	0.007 (*n* = 4)	0.082 (*n* = 4)	0.004 (*n* = 4)	0.009 (*n* = 4)	0.031 (*n* = 4)	0.002 (*n* = 4)

*^a^Hippocampus was homogenized immediately after dissection from a decapitated head. This condition reflects the basal concentration of steroids in hippocampus*.

*^b^Female samples were prepared from rats at each stage of estrous cycle (Proestrus, Estrus, Diestrus1, and Diestrus 2) and ovariectomized (OVX) rats*.

*^c^Data are expressed as mean and are represented as nanomolar. Concentration in nanomolar is calculated using the average volume of 0.14 mL for one whole hippocampus that has 0.14 ± 0.02 g wet weight (*n* = 86). We assume that tissue having 1 g of wet weight has an approximate volume of 1 mL, since the major part of tissue consists of water whose 1 mL weight is 1 g (47)*.

*^d^Number of animals*.

*Modified from Ref. (37, 79, 81)*.

Surprisingly, mRNA levels of steroidogenic enzymes, including StAR, P450(17α), 17β-HSD (types 1 and 3), 5α-reductase (types 1 and 2), and P450arom, did not fluctuate in the hippocampus across the estrous cycle (79, 81). Steroid receptors, including ERα, ERβ, AR, and PR, also kept their expression level constant. Moreover, no sex difference was observed concerning these enzymes in the hippocampus whose expression levels are approximately 1/300 ~ 1/1000 of those in gonads or adrenals(79, 81, 82).

Penetration of plasma E2 into the hippocampus, however, is not able to account for the level of hippocampal E2 because hippocampal E2 is much higher than that in plasma. There are two possibilities for explanation of hippocampal E2 fluctuation. The first is the fluctuation of blood PROG which is well known to fluctuate across the estrous cycle (80). This peripherally produced PROG may penetrate hippocampus and be converted into E2, resulting in E2 fluctuation. The other is the fluctuation of activity of kinases including MAPK, Akt, and LIMK, across the estrous cycle (72, 83, 84). The activity of P450arom (E2 synthase) changes upon phosphorylation (85). If the activity of kinases fluctuates, then following the fluctuation of P450arom activity may generate hippocampal E2 fluctuation, even if the mRNA levels of steroidogenic enzymes do not change across the estrous cycle.

Female hippocampus is equipped with systems for androgen synthesis from PROG [P450(17α), 17β-HSD (types 1 and 3), 5α-reductase (types 1 and 2)] and synthesizes DHT (Table 1) (81). In female hippocampus, a large amount of Allo is also synthesized from peripherally produced PROG because 5α-reductase is responsible for Allo synthesis (53, 63, 81).

### Regulation of Local Production of Steroids in Hippocampus

A stimulation with NMDA for 30 min increases the levels of PREG and E2 in the hippocampal slices of adult male rats (42, 43, 47), suggesting that neural activity-dependent Ca^2+^ influx drives local production of PREG and E2.

Reduction of P450arom activity by phosphorylation *via* kinases (PKA and PKC) is an important mechanism which regulates E2 synthesis. Balthazart et al. demonstrated that this phosphorylation occurred in the quail brain within 15 min (85–87). In the cultured hippocampal neurons of female rats, E2 application facilitated the phosphorylation of P450arom, suggesting negative feedback mechanism (88).

As slow/genomic modulators, *cis*-retinoic acid (89) and gonadotropin-releasing hormone (GnRH) (75) were examined using hippocampal slice culture from neonatal rats. Forty-eight-hour treatment with 1 µM of 9-*cis*-retinoic acid increased the expression levels of P450(17α) and P450arom in the cultured hippocampal slices from male rats, *via* retinoid X receptor signaling (89). On the other hand, 8 days of treatment with GnRH enhanced local E2 production (75, 90). Hippocampal E2 synthesis was also increased by a stereotaxic injection of GnRH into the hippocampus of adult female rats (91).

Interestingly, behaviors, including social interaction (92, 93) and exercise (94), alter local production of steroids in the hippocampus. Social isolation (housing individually for 8 weeks) upregulated the mRNA levels of P450arom and StAR in the hippocampus of adult male rats, compared with pair housed rats (92), whereas environmental enrichment (housing in a group of nine in a large cage for 8 weeks) increased the mRNA levels of 5α-reductase type 1 and 3α-HSD (93).

## Physiological Roles of Hippocampus-Synthesized Steroids

### Hippocampus-Synthesized DHT Enhances Neurogenesis in DG

Adult hippocampal neurogenesis occurs in DG throughout life in mammals (95). Sex steroids (96–99) and exercise (100, 101) enhance adult hippocampal neurogenesis of rodents, but the involvement of sex steroids in the exercise-induced neurogenesis, had been poorly understood.

Recently, Okamoto et al. revealed that mild exercise (30 min/day for 2 weeks) increased synthesis of hippocampal DHT, resulting in the neurogenesis enhancement (94). Injection of flutamide, an AR antagonist, suppressed the exercise-induced increase in neurogenesis, suggesting the involvement of androgens. However, surprisingly, castration (depletion of androgen from blood circulation) did not suppress this effect, suggesting the involvement of hippocampus-synthesized androgens. Indeed, the increase in DHT and 5α-reductase (DHT synthase) mRNA, were observed in the hippocampus of castrated rats after exercise (94). This study provides the direct evidence of the role of hippocampus-synthesized steroids in hippocampal functions.

### Modulation of LTP/LTD Induced by Hippocampus-Synthesized Steroids

The physiological roles of hippocampus-synthesized sex steroids (E2 and DHT) in LTP/LTD were demonstrated *in vitro* studies using acute hippocampal slices and selective inhibitors of steroidogenic enzymes. A perfusion with letrozole, a selective inhibitor of P450arom, suppressed the magnitude of LTP in CA1–CA3 synapses of adult male rats (102), and in DG synapses of young (3- to 4-week old) male rats (103–104), within 10–20 min. ICI182,780, a selective antagonist of ERα/β, mimicked this suppressive effect (102), suggesting that hippocampus-synthesized E2 is required for full induction of LTP *via* synaptic ER. Conversely, hippocampus-synthesized DHT is required for the induction of LTD, from the data showing that low frequency stimulation (1 Hz, 15 min)-induced LTD was suppressed in the presence of finasteride, an inhibitor of 5α-reductase (105). In addition to sex steroids, the effect of hippocampus-synthesized PREG is reported, in which the application of aminoglutethimide, an inhibitor of P450scc, decreased the field excitatory postsynaptic potentials in granule cells in 20 min (103). Although the molecular mechanism underlying these effects remains unclear, a possible explanation may be provided by analogy from the data, showing that exogenous application of E2 rapidly (within 30 min) enhanced LTP by driving kinase network (Erk MAPK, PKA, PKC, PI3K, and CaMKII) in a non-genomic manner (27, 106).

### Role of Hippocampus-Synthesized E2 in Hippocampus-Dependent Memory

Recently, the role of hippocampus-synthesized E2 in hippocampus-dependent memory consolidation was provided using OVX mice (107). Immediate after the training, bilateral infusion of letrozole into the dorsal hippocampus blocked the transient elevation of hippocampal E2 (within 30 min), and impaired object recognition and object placement memory consolidation (107). Under the same condition except for infusion of E2, this group previously demonstrated that E2 enhanced hippocampal memory consolidation *via* rapid activation of Erk MAPK and PI3K/Akt (108, 109), suggesting that learning experience-induced E2 elevation in the hippocampus rapidly activates kinase cascades.

## Conclusion

Hippocampus-synthesized steroids as well as circulation-derived ones, are of importance for hippocampal functions. A possible molecular mechanism for rapid effect of hippocampus-synthesized steroids may be kinase networks which modulate hippocampal functions, including spinogenesis (106, 110), LTP (27), learning, and memory (108, 109).

## Author Contributions

YH wrote the manuscript. SK brushed up the initial draft of the manuscript written by YH.

## Conflict of Interest Statement

The authors declare that the research was conducted in the absence of any commercial or financial relationships that could be construed as a potential conflict of interest.
